# Darcy-Forchheimer nanofluidic flow manifested with Cattaneo-Christov theory of heat and mass flux over non-linearly stretching surface

**DOI:** 10.1371/journal.pone.0221302

**Published:** 2019-08-20

**Authors:** Ghulam Rasool, Ting Zhang

**Affiliations:** School of Mathematical Sciences, Yuquan Campus, Zhejiang University, Hangzhou, People’s Republic of China; Shandong University of Science and Technology, CHINA

## Abstract

This research article aims to disclose the features of nanofluidic flow manifested with Cattaneo-Christov model of heat and mass flux over non-linearly stretching surface. An incompressible visco-elastic nanofluid saturates the given porous medium through Darcy-Forchheimer relation. A non-uniformly induced magnetic effect is considered to accentuate the electro-magnetic and thermal conductivity of the base fluid. The model is restricted to small magnetic Reynolds. Boundary layer assumptions are incorporated for the given flow model. Governing equations are remodeled into non-linear ordinary differential equations through transformations. So formulated nonlinear system is solved through homotopy analysis method (HAM) to achieve series solutions for velocity field, concentration of nanoparticles and temperature distribution. It is noticed that the temperature distribution and corresponding thermal boundary layer pattern shows declination for Cattaneo-Christov model of heat and mass flux as compared to classical Fourier’s law of heat flux/conduction. Furthermore, the intensive resistance offered by the addition of porosity factor in the flow model results in rise of temperature profile, however, opposite behavior is noticed in concentration of nanoparticles. The wall-drag intensity, the heat flux and the mass flux are discussed on the premise of numerical information obtained upon simulation of the problem.

## 1 Introduction

Due to considerable importance of nanofluids in industrial, chemical, environmental, geological and may other setups, the concept of suspension of metallic nano-size particles in typical base fluids presented by Choi [1], termed as nanofluids, has become a subject of immense interest for numerous researchers especially physicists for the past many years. The repute of nano-science in present era enforces us to interpret the behavior of fluids and nanofluids over various surfaces including stretching sheets, cylinders and plates etc. to analyze the drag force variation, heat and mass flux mechanism and other important industrial aspects (see for example [2] [3] [4] [5] [6] [7] [8] [9] [10] [11] [12] [13] [14] [15]) and cross references cited therein where authors have focused on the heat and mass flux attributes of nanofluids. Industrial and environmental setups that are likely to involve the heat and mass flux such as geological setups, chemical actor-reactors and other heating systems are the main focus in convection through porous medium. The porosity factor involved in such systems is merely to induce the incremental inertial effect that results in variation of skin friction (wall-drag). Such formulations are helpful in building heat insulation related materials, energy storage setups, fossil fuel beds, disposal reactors for nuclear wastes and many others. The concept was initially introduced by Darcy and the law, known as Darcy’s law, is valid for a restricted range of velocity. In the case of porous media, it becomes almost impossible to ignore the high effects of inertial force and resistance offered by the porosity factor. Thus, a squared velocity term within the context of Darcian velocity is involved in the momentum equation to analyze the inertial effects. Numerous articles have been reported on this subject explaining various influential parameters. For example, Hayat et al. [16] reported Darcy model flow using Cattaneo-Christov theory of heat and mass flux over a linearly stretching sheet. Reportedly, the momentum of the fluid and the associated boundary layer resulted a declination for augmented values of porosity factor. In another article, Bakar et al. [17] disclosed the fluid flow analysis at stagnation point. The fluid momentum and the thermal boundary layer resulted in reducing behavior for augmented porosity factor. Taseer et al. [18] has introduced a revised model for Maxwell nanofluid using Darcy Forchheimer relation. Similar results have been reported in this study as compared to [17]. Alshomrani et al. [19] involved a convectively heated stretching surface manifested with Darcy flow model to analyze the impact of porosity and enhanced frictional force on fluid flow. Mandal and Seth [20] reported a Casson type nanofluid flow with Darcy-Forchheimer relation. The magnetic effect resulted in obvious declination of the fluid momentum. Hayat et al. [21] implemented Darcy model for curvy stretching sheet witnessing an increase in thermal layer for incremental nature of porosity factor. Recently, Sheikholeslami et al. [22] reported the impact of MHD (Lorentz forces) on *Fe*_3_*O*_4_-*H*_2_*O* Ferro-fluid flow using permeable semi-annulus. The porosity factor resulted in higher resistance offered to the fluid motion following a low velocity field. In another article, Sheikholeslami et al. [23] analyzed the behavior of water based nanofluid in a porous media/enclosure with quite similar results. Dogonchi et al. [24] reported a *Cu* − *H*_2_*O* based nanofluid flow through porous media between a hot and cold cylindrical framework. The impact of thermal radiation and porosity factor in *CuO* − *H*_2_*O* based nanofluid has been analyzed by Dogonchi et al. [25]. A novel correlation for the averaged Nusselt number has been presented in this study. Quite similar but more effective results have been reported in the study presented by Dogonchi et al. [26] involving an annulus subject to thermal radiation.

Recently, the engagement of Cattaneo-Christov model in nanofluids to analyze the heat and mass flux mechanism is trending as numerous researchers are working on such formulations. Heat flux is a natural process that occurs where there is a temperature difference within a system or within the systems. Usually, the law of thermodynamics and heat conduction reported by Fourier [27] has been extensively used for many years to analyze the heat and mass characteristics however, it restricts the energy and concentration equations to parabolic type equations that means an initial disturbance would lead to instant experience by the system, called a paradox of heat and mass flux. This restriction was removed by Cattaneo [28] by enforcing a modification with relaxation time factor. This term, therefore, covers/overcomes the heat and mass flux paradox. Later on, Christov [29] further improved the version of model presented by Cattaneo by replacing time derivative with Oldroyd upper-convective derivative. The theory, thus, termed as Cattaneo-Christov heat and mass flux theory (CC-model). Related to this discussion, Haddad [30] analyzed thermal instability using CC-model in Brinkman porous medium. Li et al. [31] utilized CC-model in heat and mass flux analysis of visco-elastic fluid flow over stretching surface subject to slip conditions. A decreasing velocity profile is noticed for enhanced magnetic effect however, the result is opposite for the thermal profile. Sui et al. [32] disclosed the influence of CC-model on Maxwell nanofluidic flow through stretching surface subject to slip conditions. Relatively small impact of relaxation parameters is noticed on velocity profile as compared to thermal and salute layers. Ganji and Dogonchi [33] reported a squeezing flow of nanofluids confined in parallel plates involving CC-model and thermal radiation. The results indicated that thermal distribution is lesser for CC-model as compared to the classical Fourier’s law. Upadhay et al. [34] discussed the impact of CC-model on heat and mass transfer attributes in Powell-Eyring type nanofluidic flow. The thermal relaxation time parameter resulted in an improvement in heat flux rate. Acharya et al. [35] reported impact of CC-model in a magnetized upper convective Maxwell type nanofluidic flow past an inclined stretching sheet. The study was conducted in the context of generalized Fourier and Ficks perspectives.

The theory of fluid flow, heat and mass transfer analysis over a stretching sheet/surface and porous media is an important aspect. It’s numerous applications in engineering procedures like paper production, aluminous plate cooling procedures, plastic sheet extrusions etc. are remarkably best-known to the readers. This immense interest in the involvement of stretching sheet in fluid flow resulted in numerous research studies reported in the last two decades. For example, Sajid et al. [36] mentioned viscous flow of steady incompressible fluid instigated by curvy extended stretching sheet. It is reported that the wall-drag force (skin-friction) on a curvy surface is lesser than a flat surface. The studies mentioned above apprehend the argument that more focus has been emphasized on linear stretching surfaces and non-linearity in stretching rate has been neglected upto a decent level though, the stretching with nonlinear pattern is reasonably important in practical applications of fluids/nanofluids. The concept is new maneuver and trending recently. Therefore, keeping in view the context of non-linearity in stretching rates, recently, Rasool et al. [37] presented MHD nanofluidic flow over non-linearly stretching surface/sheet reporting some interesting jumps in the heat and mass flux rates. An innovative study on MHD nanofluidic flow over vertically stretching surface/sheet has been reported by Alarifi et al. [38]. The model involved heat source to balance the base temperature of the surface. A report on second grade nanofluid flow past a porous media has be presented by Khan et al. [39]. The study incorporated stretching surface/sheet, heat and mass transfer attributes. In a similar study, Khan et al. [40] reported an analysis on heat-mass flux attributes as well as thermophoresis and Brownian diffusion in MHD nanofluid flow over stretching surface. Palwasha et al. [41] reported a non-Newtonian nanofluid flow through porous media using microorganisms magneto-tactics. Some recent studies can be seen in ([42] [43] [44] [45] [46]) and cross references cited therein.

The MHD (Magnetohydrodynamics) is another very important aspect in engineering, chemical and environmental setups involving fluid flow analysis. Instruments such as bearings, generators, chemical actor/reactors, pumps and many others are tormented by MHD. The applications of MHD are also found in industrial systems where a high speed machine is working having tiny size. The temperature range is witnessed between zero to 350-degrees on the scale of Celcius. Over the years, the concept of MHD has been involved in fluid flow analysis to help improve the thermal and electro-magnetic conductivity of the subjected fluid/nanofluid. Some related studies have been reported in this text. For example, Singh et al. [47] presented an MHD flow using variable thermal conductivity. The analysis involved stagnation point formulation. MHD convective heat-mass transfer under Soret-Dufour effects has been reported by Chatterjee et al. [48] using Power-Law model and porosity. Lund et al. [49] reported a study on the dual solution for MHD Williamson fluid flow with slippage. Khan et al. [50] reported MHD mixed convection in second grade nanofluidic flow considering Browniian diffusion and thermophoresis together with Hall effects over a stretching sheet/surface. Khan et al. [51] involved MHD in nanoliquid thin film flow in a cylinder. Zuhra et al. [52] discussed the heat-mass flux attributes in second grade MHD nanofluid flow saturated with gyrotactic micro-organisms and nanoparticles. The influence of inclined MHD on heat and mass transfer attributes in Carreau nanoliquid flow has been reported by Khan et al. [53]. MHD *Cu* − *H*_2_*O* based natural convection in complex shaped enclosure has been reported by Dogonchi et al. [54]. A similar study on copper-water nanofluidic flow through a horizontal semi-cylinder has been reported by Dogonchi et al. [55].

In all the studies mentioned above, the major emphasis has been given to linearity in stretching rates. Here in this research, the non-linearity in stretching rate of the surface has been targeted to interpret various aspects of fluid flow analysis. Furthermore, no such study is found in literature involving non-linear stretching surface, Darcy-Forchheimer relation and Cattaneo-Christov model of heat-mass flux all together. The article is organized as follows: Firstly, a Darcy-Forchheimer relation along with Cattaneo-Christov model of heat and mass flux is implemented on steady, incompressible and viscoelastic nanofluid flow bounded by a flat non-linearly stretching sheet/surface. Boundary layer assumptions, Brownian diffusion and thermophoresis are attended. Secondly, the governing equations are remodeled into non-linear ordinary differential equations through transformations. Thirdly, HAM [56] is used to get the final series solutions. Fourthly, graphs are plotted to investigate the variation in velocity field, temperature distribution and concentration of the nanoparticles. Finally, a correlation is given for elevated values of different parameters to help the audience in understanding the variation in skin-friction (wall-drag), heat and mass flux rates.

## 2 Problem formulation

**Fig 1:**
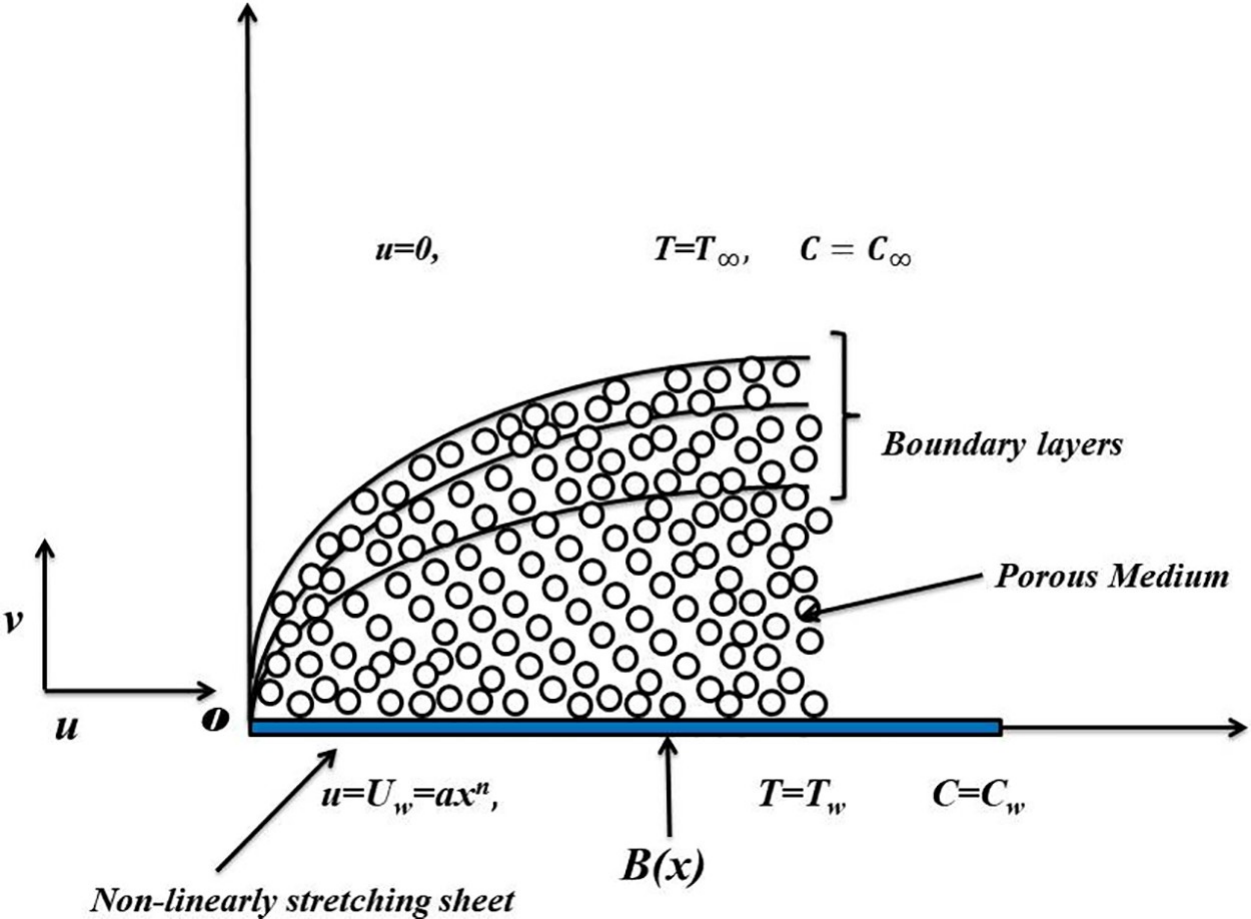
Physical model and coordinate system.

Consider a viscoelastic incompressible nanofluidic flow manifested with Cattaneo-Christov model of heat and mass flux over non-linearly stretching surface. The nanofluid saturates the given porous medium through Darcy-Forchheimer relation. A non-uniformly induced magnetic effect is involved to accentuate the electro-magnetic and thermal conductivity of the base fluid. The model is restricted to small Reynolds to dismiss the influence of induced magnetic effect. Cattaneo-Christov model is employed to involve the effect of modified Fourier’s law. The stretching velocity is *u* = *U*_*w*_ = *ax*^*n*^ where *n* = 1 implies linear rate of stretching and *n* > 1 implies non-linearity in stretching of the sheet. The stretching rate is assumed to be non-linear for the given flow model. The sheet extends along *x*–direction while *y*–direction is taken surface normal to it. There is no fluid movement along the *y*–axis and the steady flow is assumed along *x*–axis only. Schematic can be seen in Fig 1.

The governing equations are as follows,

$$\begin{array}{c} \frac{\partial v}{\partial y}+\frac{\partial u}{\partial x}=0, \end{array}$$ (1)

$$\begin{array}{c} \begin{array}{cc} v\frac{\partial u}{\partial y}+u\frac{\partial u}{\partial x}= & \nu \frac{\partial^{2}u}{\partial y^{2}}-\frac{C_{b}}{K^{1/2}}u^{2}-[\frac{\sigma B_{0}^{2}{\left(x\right)}^{n-1}}{\rho_{fl}}-\frac{\nu }{K}]u\\ & -k_{0}(u\frac{\partial^{3}u}{\partial x\partial y^{2}}+v\frac{\partial }{\partial y}(\frac{\partial^{2}u}{\partial y^{2}})+\frac{\partial u}{\partial x}\frac{\partial^{2}u}{\partial y^{2}}-\frac{\partial^{2}u}{\partial x\partial y}\frac{\partial u}{\partial y}), \end{array} \end{array}$$ (2)

$$\begin{array}{c} \rho c_{p}(u\frac{\partial T}{\partial x}+v\frac{\partial T}{\partial y})=-\nabla .\vec{p}, \end{array}$$ (3)

$$\begin{array}{c} \rho c_{p}(u\frac{\partial C}{\partial x}+v\frac{\partial C}{\partial y})=-\nabla .\vec{q}. \end{array}$$ (4)

According to Cattaneo-Charistov heat flux theory, see for example [35], we have,

$$\begin{array}{c} \vec{p}=-k\nabla T-\lambda_{1}(\frac{\partial \vec{p}}{\partial t}+\vec{v}\cdot \nabla \vec{p}-\vec{p}\cdot \nabla \vec{v}+(\nabla \cdot \vec{v})\vec{p}), \end{array}$$ (5)

and

$$\begin{array}{c} \vec{q}=-D_{Br}\nabla C-\lambda_{2}(\frac{\partial \vec{q}}{\partial t}+\vec{v}\cdot \nabla \vec{q}-\vec{q}\cdot \nabla \vec{v}+(\nabla \cdot \vec{v})\vec{q}). \end{array}$$ (6)

(5) & (6) are the equations for heat and mass flux with relaxation time λ_1_ for thermal and λ_2_ for solute levels. Classical Fourier’s law can be deduced by setting λ_1_ = λ_2_ = 0 in the above mentioned equations. Considering the natural incompressibility i.e. $\nabla \cdot \vec{v}=0$ and assumption of steady flow i.e. $\frac{\partial p}{\partial t}=\frac{\partial q}{\partial t}=0$ in (5-6) yields,

$$\begin{array}{c} \vec{p}=-k\nabla T-\lambda_{1}(\vec{v}\cdot \nabla \vec{p}-\vec{p}\cdot \nabla \vec{v}), \end{array}$$ (7)

and,

$$\begin{array}{c} \vec{q}=-D_{Br}\nabla C-\lambda_{2}(\vec{v}\cdot \nabla \vec{q}-\vec{q}\cdot \nabla \vec{v}). \end{array}$$ (8)

The respective equations of energy and concentration of nanoparticles are, therefore,

$$\begin{array}{c} v\frac{\partial T}{\partial y}+u\frac{\partial T}{\partial x}+\lambda_{1}\Phi_{E}=\alpha \frac{\partial^{2}T}{\partial y^{2}}+\frac{{\left(\rho c\right)}_{np}}{{\left(\rho c\right)}_{fl}}[D_{Br}(\frac{\partial T}{\partial y}\frac{\partial C}{\partial y})+\frac{D_{Th}}{T_{\infty }}(\frac{\partial T}{\partial y}{)}^{2}], \end{array}$$ (9)

$$\begin{array}{c} v\frac{\partial C}{\partial y}+u\frac{\partial C}{\partial x}+\lambda_{2}\Phi_{S}=\frac{D_{Th}}{T_{\infty }}(\frac{\partial^{2}T}{\partial y^{2}})+D_{Br}(\frac{\partial^{2}C}{\partial y^{2}}), \end{array}$$ (10)

where,

$$\begin{array}{c} \begin{array}{cc} \Phi_{E}= & \frac{\partial T}{\partial x}(u\frac{\partial u}{\partial x})+\frac{\partial T}{\partial y}(v\frac{\partial v}{\partial y})+\frac{\partial T}{\partial y}(u\frac{\partial v}{\partial x})+\frac{\partial T}{\partial x}(v\frac{\partial u}{\partial y})+2vu\frac{\partial }{\partial y}(\frac{\partial T}{\partial y})\\ & +u^{2}\frac{\partial }{\partial x}(\frac{\partial T}{\partial x})+v^{2}\frac{\partial }{\partial y}(\frac{\partial T}{\partial y}), \end{array} \end{array}$$ (11)

and

$$\begin{array}{c} \begin{array}{cc} \Phi_{S}= & \frac{\partial C}{\partial x}(u\frac{\partial u}{\partial x})+\frac{\partial C}{\partial y}(v\frac{\partial v}{\partial y})+\frac{\partial C}{\partial y}(u\frac{\partial v}{\partial x})+\frac{\partial C}{\partial x}(v\frac{\partial u}{\partial y})+2vu\frac{\partial }{\partial y}(\frac{\partial C}{\partial y})\\ & +u^{2}\frac{\partial }{\partial x}(\frac{\partial C}{\partial x})+v^{2}\frac{\partial }{\partial y}(\frac{\partial C}{\partial y}), \end{array} \end{array}$$ (12)

with boundary conditions,

$$\begin{array}{c} u=U_{w}=ax^{n},\;C=C_{w},\;v=0,\;T=T_{w}\;\;\text{at}\;\;y=0, \end{array}$$ (13)

$$\begin{array}{c} C=C_{\infty },\;u=0,\;T=T_{\infty }\;\;\text{as}\;\;y\to \infty . \end{array}$$ (14)

Defining,

$$\begin{array}{c} \begin{array}{cc} & \;v=-\frac{1}{2}\sqrt{2a\nu (n+1)}x^{\frac{n-1}{2}}[f\left(\eta \right)+(\frac{n-1}{n+1})\frac{\partial f}{\partial \eta }\eta ],\;u=ax^{n}\frac{\partial f}{\partial \eta },\\ & \theta \left(\eta \right)=\frac{T-T_{\infty }}{T_{w}-T_{\infty }},\;\;\phi \left(\eta \right)=\frac{C-C_{\infty }}{C_{w}-C_{\infty }},\;\;\eta =\frac{1}{2}\sqrt{\frac{2\rho_{fl}a\left(n+1\right)}{\mu }}x^{\frac{n-1}{2}}y. \end{array} \end{array}$$ (15)

Using (15) in (1)–(2) & (9)–(10), we have

$$\begin{array}{c} \begin{array}{cc} & f^{\prime \prime \prime }+ff^{\prime \prime }-\lambda f^{\prime }-M^{2}f^{\prime }-k_{1}[(\frac{n+1}{2n})f^{\prime }f^{\prime \prime \prime }-(\frac{n+1}{2n})f^{\prime }f^{iv}-(f^{\prime \prime }{)}^{2}]\\ & -(\frac{2n}{1+n})(1+F_{r})(f^{\prime }{)}^{2}=0, \end{array} \end{array}$$ (16)

$$\begin{array}{c} \begin{array}{cc} & \frac{1}{Pr}\theta^{\prime \prime }+f\theta^{\prime }-\gamma_{1}[(\frac{n-1}{2})ff^{\prime }\theta^{\prime }+(\frac{n+1}{2})f^{2}\theta^{\prime \prime }]+Nb\theta^{\prime }\phi^{\prime }+Nt(\theta^{\prime }{)}^{2}=0, \end{array} \end{array}$$ (17)

$$\begin{array}{c} \begin{array}{cc} & \phi^{\prime \prime }+LePrf\phi^{\prime }-\gamma_{2}LePr[(\frac{n-1}{2})ff^{\prime }\phi^{\prime }+(\frac{n+1}{2})f^{2}\phi^{\prime \prime }]+\frac{Nt}{Nb}\theta^{\prime \prime }=0, \end{array} \end{array}$$ (18)

$$\begin{array}{c} \begin{array}{cc} & f\left(0\right)=0,\;\phi \left(0\right)=1,\;\theta \left(0\right)=1,\;f^{\prime }\left(0\right)=1,\\ & f^{\prime }\left(\infty \right)=0,\;\phi \left(\infty \right)=0,\;\theta \left(\infty \right)=0. \end{array} \end{array}$$ (19)

Here, *γ*_*i*_ for *i* = 1, 2 is the relaxation time parameter for temperature and concentration of nanoparticles. *F*_*r*_ is local inertial force, *M* is magnetic effect, λ is known for porosity, *Pr* is known as Prandtl factor, *Le* is known as Lewis factor, *k*_1_ is known as viscoelastic factor, *Nb* is called Brownian diffusion factor and *Nt* is called thermophoretic force factor. Mathematically,

$$\begin{array}{c} \begin{array}{cc} & \gamma_{i}=ax^{n-1},\;F_{r}=\frac{2C_{b}x}{K^{1/2}\left(n+1\right)},\;M^{2}=\frac{2\sigma B_{0}^{2}}{a\rho_{f}\left(n+1\right)},\\ & \lambda =\frac{2\nu }{Ka\left(n+1\right)x^{n-1}},\;\mathrm{Pr}=\frac{\nu }{\alpha },\;Le=\frac{\alpha }{D_{Br}},\;k_{1}=\frac{k_{0}ax^{n-1}}{\nu },\\ & Nb=\frac{{\left(\rho_{fl}c\right)}_{np}D_{Br}\left(C_{w}-C_{\infty }\right)}{{\left(\rho_{fl}c\right)}_{fl}\nu },\;Nt=\frac{{\left(\rho_{fl}c\right)}_{np}D_{Th}(T_{w}-T_{\infty })}{{\left(\rho_{fl}c\right)}_{fl}\nu T_{\infty }}. \end{array} \end{array}$$ (20)

Quantities of physical and industrial interest such as wall drag (*C*_*f*_), heat flux (local Nusselt) (*Nu*) and mass flux (local Sherwood) (*Sh*) numbers are given by:

$$\begin{array}{c} \begin{array}{c} C_{f}=\frac{1}{\rho U_{w}^{2}}\left(\tau_{w}\right), \end{array}\\ \begin{array}{c} Nu_{x}=\frac{x}{k\left(T_{w}-T_{\infty }\right)}\left(q_{w}\right), \end{array}\\ Sh_{x}=\frac{x}{D_{Br}\left(C_{w}-C_{\infty }\right)}\left(q_{m}\right), \end{array}$$ (21)

where *q*_*w*_, *τ*_*w*_ and *q*_*m*_ are the surface heat flux, shear stress and surface mass flux, respectively. Using the definitions of *τ*_*w*_, *q*_*w*_ and *q*_*m*_ and simplifying,

$$\begin{array}{c} C_{f}=\frac{1}{U_{w}^{2}}\left(v\frac{\partial u}{\partial y}-k_{0}{\left(v\frac{\partial^{2}u}{\partial y^{2}}+u\frac{\partial^{2}u}{\partial x\partial y}-2\frac{\partial u}{\partial y}\frac{\partial v}{\partial y}\right)}_{y=0}\right),\\ \begin{array}{c} Nu_{x}=\frac{x}{\left(T_{\infty }-T_{w}\right)}\left[\left(\frac{\partial }{\partial y}{\left(T\right)}_{y=0}\right)\right], \end{array}\\ \begin{array}{c} Sh_{x}=\frac{x}{\left(C_{\infty }-C_{w}\right)}\left[\left(\frac{\partial }{\partial y}{\left(C\right)}_{y=0}\right)\right]. \end{array} \end{array}$$ (22)

Therefore, the non-dimensional forms are given below:

$$\begin{array}{c} \begin{array}{c} {\left(Re_{x}\right)}^{1/2}C_{f}={\left(\frac{n+1}{2}\right)}^{1/2}\left[\left(1-3k_{1}\right)f^{\prime \prime }\left(0\right)\right], \end{array}\\ {\left(Re_{x}\right)}^{-1/2}Nu=-{\left(\frac{n+1}{2}\right)}^{1/2}\left[\theta^{\prime }\left(0\right)\right],\;{\left(Re_{x}\right)}^{-1/2}Sh=-{\left(\frac{n+1}{2}\right)}^{1/2}\left[\phi^{\prime }\left(0\right)\right], \end{array}$$ (23)

where *Re*_*x*_ = *ax*^*n*+1^/*ν* is the local Reynolds number.

## 3 Methodology

Homotopy analysis method (HAM) is an efficient solver for nonlinear systems where small and large physical parameters are involved for analysis of variation in the respective flow profiles. Thus, it is more suitable and economical as compared to that of perturbation method for solving non-linear systems of equations. It is applied in most of non-linear systems developed in engineering, industrial, science and finance problems. Define,

$$\begin{array}{c} f_{0}=1-\exp\left(-\eta \right),\;\;\theta_{0}=\exp\left(-\eta \right),\;\;\phi_{0}=\exp\left(-\eta \right), \end{array}$$ (24)

$$\begin{array}{c} {\hat{L}}_{f}=\frac{\partial^{3}f}{\partial \eta^{3}}-\frac{\partial f}{\partial \eta },\;\;{\hat{L}}_{\theta }=\frac{\partial^{2}\theta }{\partial \eta^{2}}-\theta ,\;\;{\hat{L}}_{\phi }=\frac{\partial^{2}\phi }{\partial \eta^{2}}-\phi , \end{array}$$ (25)

such that,

$$\begin{array}{c} \begin{array}{cc} & {\hat{L}}_{f}[K_{1}e^{-\eta }+K_{2}e^{\eta }+K_{3}]=0,\;\;{\hat{L}}_{\theta }[K_{4}e^{-\eta }+K_{5}e^{\eta }]=0,\;\;{\hat{L}}_{\phi }[K_{6}e^{-\eta }+K_{7}e^{\eta }]=0, \end{array} \end{array}$$ (26)

where *K*_*i*_, *i* = 1, 2, ⋯, 7, are constants. Subsequently, the *zeroth* order deformation problems:

$$\begin{array}{c} \begin{array}{cc} P_{f}[\hat{f}] & =\frac{\partial^{3}\hat{f}}{\partial^{3}\eta }+\hat{f}(\frac{\partial {\hat{f}}^{2}}{\partial^{2}\eta })-M^{2}\frac{\partial \hat{f}}{\partial \eta }-\lambda \frac{\partial \hat{f}}{\partial \eta }-[\frac{2n}{n+1}](1+F_{r})(\frac{\partial \hat{f}}{\partial \eta }{)}^{2}\\ & -k_{1}[(\frac{n+1}{2n})\frac{\partial \hat{f}}{\partial \eta }\frac{\partial^{3}\hat{f}}{\partial \eta^{3}}-(\frac{n+1}{2n})\frac{\partial \hat{f}}{\partial \eta }\frac{\partial^{4}\hat{f}}{\partial \eta^{4}}-(\frac{\partial^{2}\hat{f}}{\partial \eta^{2}}{)}^{2}], \end{array} \end{array}$$ (27)

$$\begin{array}{c} \begin{array}{cc} P_{\theta }[\hat{f},\hat{\theta },\hat{\phi }] & =\frac{1}{Pr}\frac{\partial^{2}\hat{\theta }}{\partial \eta^{2}}+\hat{f}(\frac{\partial \hat{\theta }}{\partial \eta })\\ & -\gamma_{1}[(\frac{n-1}{2})\hat{f}\frac{\partial \hat{f}}{\partial \eta }\frac{\partial \hat{\theta }}{\partial \eta }+(\frac{n+1}{2}){\hat{f}}^{2}\frac{\partial^{2}\hat{\theta }}{\partial \eta^{2}}]+Nt(\frac{\partial \hat{\theta }}{\partial \eta }{)}^{2}+Nb\frac{\partial \hat{\theta }}{\partial \eta }\frac{\partial \hat{\phi }}{\partial \eta }, \end{array} \end{array}$$ (28)

$$\begin{array}{c} \begin{array}{cc} P_{\phi }[\hat{f},\hat{\theta },\hat{\phi }] & =\frac{\partial^{2}\hat{\phi }}{\partial \eta^{2}}+LePr\hat{f}(\frac{\partial \hat{\phi }}{\partial \eta })\\ & -\gamma_{2}LePr[(\frac{n-1}{2})\hat{f}\frac{\partial \hat{f}}{\partial \eta }\frac{\partial \hat{\phi }}{\partial \eta }+(\frac{n+1}{2}){\hat{f}}^{2}\frac{\partial^{2}\hat{\phi }}{\partial \eta^{2}}]+\frac{Nt}{Nb}(\frac{\partial^{2}\hat{\theta }}{\partial \eta^{2}}), \end{array} \end{array}$$ (29)

correspond to,

$$\begin{array}{c} \begin{array}{cc} & \left(1-z\right){\hat{L}}_{f}[\hat{f}\left(\eta ,z\right)-f_{0}\left(\eta \right)]=z{\hat{h}}_{f}P_{f}\left[\hat{f}\right],\\ & \left(1-z\right){\hat{L}}_{\theta }[\hat{\theta }\left(\eta ,z\right)-\theta_{0}\left(\eta \right)]=z{\hat{h}}_{\theta }P_{\theta }\left[\hat{f},\hat{\theta },\hat{\phi }\right],\\ & \left(1-z\right){\hat{L}}_{\phi }[\hat{\phi }\left(\eta ,z\right)-\phi_{0}\left(\eta \right)]=z{\hat{h}}_{\phi }P_{\phi }\left[\hat{f},\hat{\theta },\hat{\phi }\right]. \end{array} \end{array}$$ (30)

with boundary conditions,

$$\begin{array}{c} \begin{array}{cc} & \hat{f}\left(0,z\right)=0,\;\;\hat{\theta }\left(0,z\right)=1,\;\;\hat{\phi }\left(0,z\right)=1,\;\;\frac{\partial \hat{f}}{\partial \eta }{|}_{\left(0,z\right)}=1,\\ & \frac{\partial \hat{f}}{\partial \eta }{|}_{\left(\infty ,z\right)}=0,\;\;\hat{\theta }\left(\infty ,z\right)=0,\;\;\hat{\phi }\left(\infty ,z\right)=0, \end{array} \end{array}$$ (31)

where ${\hat{h}}_{f},{\hat{h}}_{\theta }$ and ${\hat{h}}_{\phi }$ are auxiliary-parameters and *z* ∈ [0, 1] is known as embedding factor. $P_{\hat{f}},P_{\hat{\theta }}$ and $P_{\hat{\phi }}$ are non-linear operators. The Taylor’s series expansion results in,

$$\begin{array}{c} \hat{f}=\sum_{i=0}^{\infty }f_{i}\left(\eta \right)z^{i},\;\;\hat{\theta }=\sum_{i=0}^{\infty }\theta_{i}\left(\eta \right)z^{i},\;\;\hat{\phi }=\sum_{i=0}^{\infty }\phi_{i}\left(\eta \right)z^{i}, \end{array}$$ (32)

where $Z_{i}\left(\eta \right)=\frac{1}{i!}\frac{\partial^{i}Z}{\partial z^{i}}{|}_{z=0}$ for $Z=\hat{f},\hat{\theta }$ or $\hat{\phi }$. The speedy and smooth convergence of the series solutions is strictly dependent on choice $\hat{h}$. For *z* = 0, 1,

$$\begin{array}{c} \begin{array}{cc} \sum_{i=0}^{\infty }f_{i}\left(\eta \right) & =f=f_{0}+\sum_{i=1}^{\infty }f_{i},\\ \sum_{i=0}^{\infty }\theta_{i}\left(\eta \right) & =\theta =\theta_{0}+\sum_{i=1}^{\infty }\theta_{i},\\ \sum_{i=0}^{\infty }\phi_{i}\left(\eta \right) & =\phi =\phi_{0}+\sum_{i=1}^{\infty }\phi_{i}. \end{array} \end{array}$$ (33)

Consequently, the *i*^*th*^ deformations:

$$\begin{array}{c} \begin{array}{cc} M_{f}[\hat{f}] & =\frac{\partial^{3}f_{i-1}}{\partial \eta^{3}}+\sum_{j=0}^{i-1}f_{i-1-j}\frac{\partial^{2}f_{j}}{\partial \eta^{2}}-M^{2}\frac{\partial f_{i-1}}{\partial \eta }\\ & -\lambda \frac{\partial f_{i-1}}{\partial \eta }-(\frac{2n}{n+1})(1+F_{r})\sum_{j=0}^{i-1}\frac{\partial f_{i-1-j}}{\partial \eta }\frac{\partial f_{j}}{\partial \eta }\\ & -k_{1}[(\frac{n+1}{2n})\sum_{j=0}^{i-1}\frac{\partial f_{i-1-j}}{\partial \eta }\frac{\partial^{3}f_{j}}{\partial \eta^{3}}-(\frac{n+1}{2n})\sum_{j=0}^{i-1}\frac{\partial f_{i-1-j}}{\partial \eta }\frac{\partial^{4}f_{j}}{\partial \eta^{4}}-(\frac{\partial^{2}f_{i}}{\partial \eta^{2}}{)}^{2}], \end{array} \end{array}$$ (34)

$$\begin{array}{c} \begin{array}{cc} M_{\theta }[\hat{f},\hat{\theta },\hat{\phi }] & =\frac{\partial^{2}\theta_{i-1}}{\partial \eta^{2}}+Pr\sum_{j=0}^{i-1}f_{i-1-j}\frac{\partial \theta_{j}}{\partial \eta }+PrNb\sum_{j=0}^{i-1}\frac{\partial \theta_{i-1-j}}{\partial \eta }\frac{\partial \phi_{j}}{\partial \eta }+PrNt\sum_{j=0}^{i-1}\frac{\partial \theta_{i-1-j}}{\partial \eta }\frac{\partial \theta_{j}}{\partial \eta }\\ & -Pr(\gamma_{1}[(\frac{n-1}{2})\sum_{j=0}^{i-1}f_{i-1-j}^{2}\sum_{k=0}^{j}\frac{\partial f_{k-j}}{\partial \eta }\frac{\partial \theta_{j}}{\partial \eta }+(\frac{n+1}{2})\sum_{j=0}^{i-1}f_{i-1-j}^{2}\frac{\partial^{2}\theta_{j}}{\partial \eta^{2}}]), \end{array} \end{array}$$ (35)

$$\begin{array}{c} \begin{array}{cc} M_{\phi }[\hat{f},\hat{\theta },\hat{\phi }] & =\frac{\partial^{2}\phi_{i-1}}{\partial \eta }+PrLe\sum_{j=0}^{i-1}f_{i-1-j}\frac{\partial \phi_{j}}{\partial \eta }-\frac{Nt}{Nb}\frac{\partial^{2}\theta_{i-1}}{\partial \eta^{2}}\\ & -PrLe\gamma_{2}[(\frac{n-1}{2})\sum_{j=0}^{i-1}f_{i-1-j}^{2}\sum_{k=0}^{j}\frac{\partial f_{k-j}}{\partial \eta }\frac{\partial \phi_{j}}{\partial \eta }+(\frac{n+1}{2})\sum_{j=0}^{i-1}f_{i-1-j}^{2}\frac{\partial^{2}\phi_{j}}{\partial \eta^{2}}], \end{array} \end{array}$$ (36)

correspond to,

$$\begin{array}{c} \begin{array}{cc} L_{f}[f_{i}-\Phi_{i}f_{i-1}] & ={\hat{h}}_{f}M_{f}^{i},\\ L_{\theta }[\theta_{i}-\Phi_{i}\theta_{i-1}] & ={\hat{h}}_{\theta }M_{\theta }^{i},\\ L_{\phi }[\phi_{i}-\Phi_{i}\phi_{i-1}] & ={\hat{h}}_{\phi }M_{\phi }^{i}, \end{array} \end{array}$$ (37)

with boundary conditions,

$$\begin{array}{c} \begin{array}{cc} & \hat{f_{i}}\left(0\right)=0,\;\;\hat{\theta_{i}}\left(0\right)=0,\;\;\hat{\phi_{i}}\left(0\right)=0,\;\;\frac{\partial \hat{f_{i}}}{\partial \eta }{|}_{\left(0\right)}=0,\\ & \frac{\partial \hat{f_{i}}}{\partial \eta }{|}_{\left(\infty \right)}=0,\;\;\hat{\theta_{i}}\left(\infty \right)=0,\;\;\hat{\phi_{i}}\left(\infty \right)=0, \end{array} \end{array}$$ (38)

where Φ_*i*_ = 1 for *i* > 1, otherwise 0. Finally,

The general solutions are,

$$\begin{array}{c} \begin{array}{cc} & f_{i}=K_{1}+K_{2}e^{\eta }+K_{3}e^{-\eta }+f_{i}^{⋆}\left(\eta \right),\\ & \theta_{i}=K_{4}e^{\eta }+K_{5}e^{-\eta }+\theta_{i}^{⋆}\left(\eta \right),\\ & \phi_{i}=K_{6}e^{\eta }+K_{7}e^{-\eta }+\phi_{i}^{⋆}\left(\eta \right), \end{array} \end{array}$$ (39)

where $f_{i}^{⋆},\theta_{i}^{⋆}$ and $\phi_{i}^{⋆}$ are special solutions.

## 4 Convergence analysis

**Fig 2:**
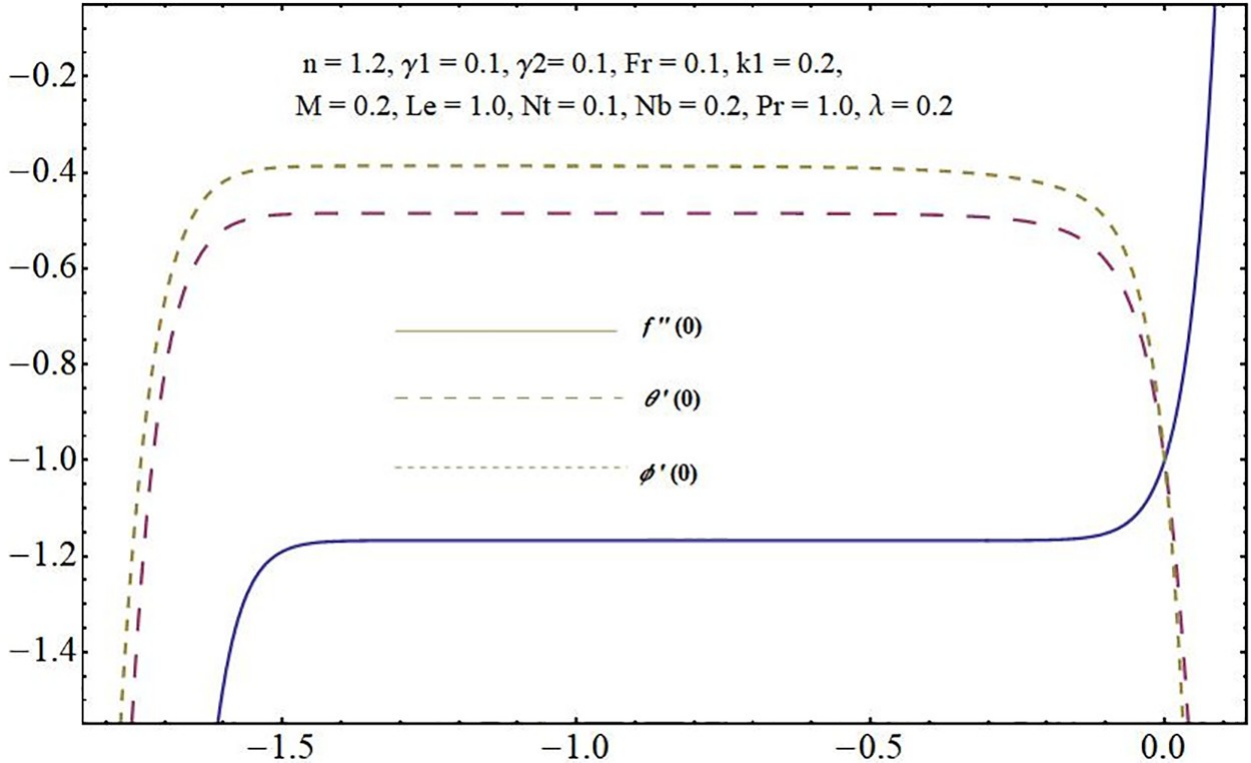
H-Curves.

The auxiliary parameters involved in series solutions for the velocity field (*f*′), temperature distribution (*θ*) and concentration of the nanoparticles (*ϕ*) for the problem under consideration are termed as convergence control parameters. These parameters are critical in choosing appropriate values to speed-up the convergence. The intervals of interest for *f*, *θ* and *ϕ* are presented in Fig 2. One can see that the intervals of convergence are [-1.30, -0.10], [-1.50, -0.10] and [-1.50, -0.10], respectively. Data upto 40th order approximations is listed in Table 1. 15^*th*^ order of approximations are sufficient to achieve convergence in velocity field whereas temperature and concentration requires 10^*th*^ order approximations.

**Table 1:** Convergence.

Approximation Order	−*f*″	−*θ*′	−*ϕ*′
1	0.779232	0.63500	0.50000
2	0.652004	0.59794	0.50578
5	0.571883	0.59486	0.50658
10	0.518020	0.59364	0.50735
15	0.452200	0.59364	0.50735
20	0.452200	0.59364	0.50735
30	0.452200	0.59364	0.50735
40	0.452200	0.59364	0.50735

## 5 Results & discussion

**Fig 3:**
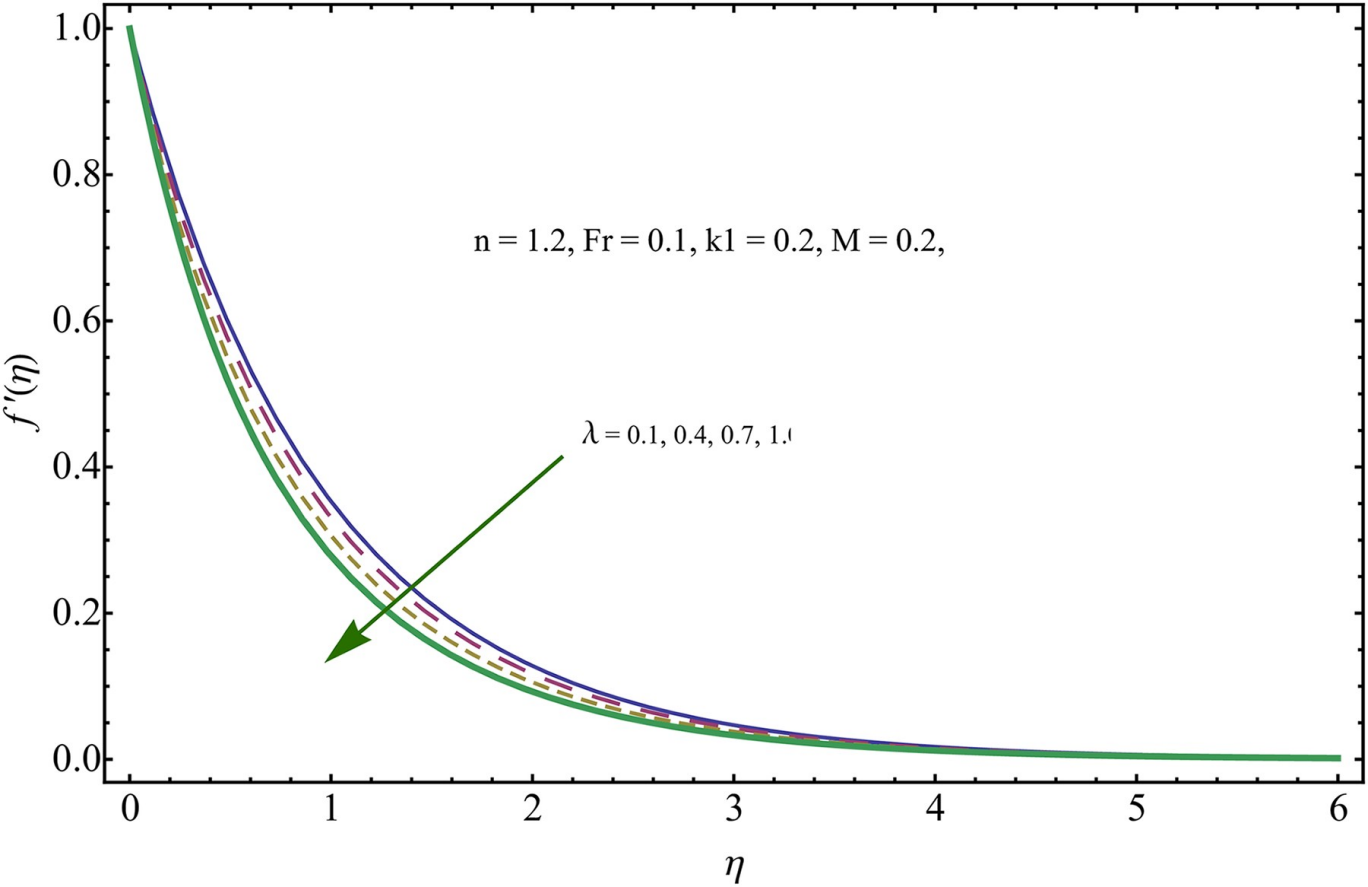
Variation in velocity field for incremental values of λ.

**Fig 4:**
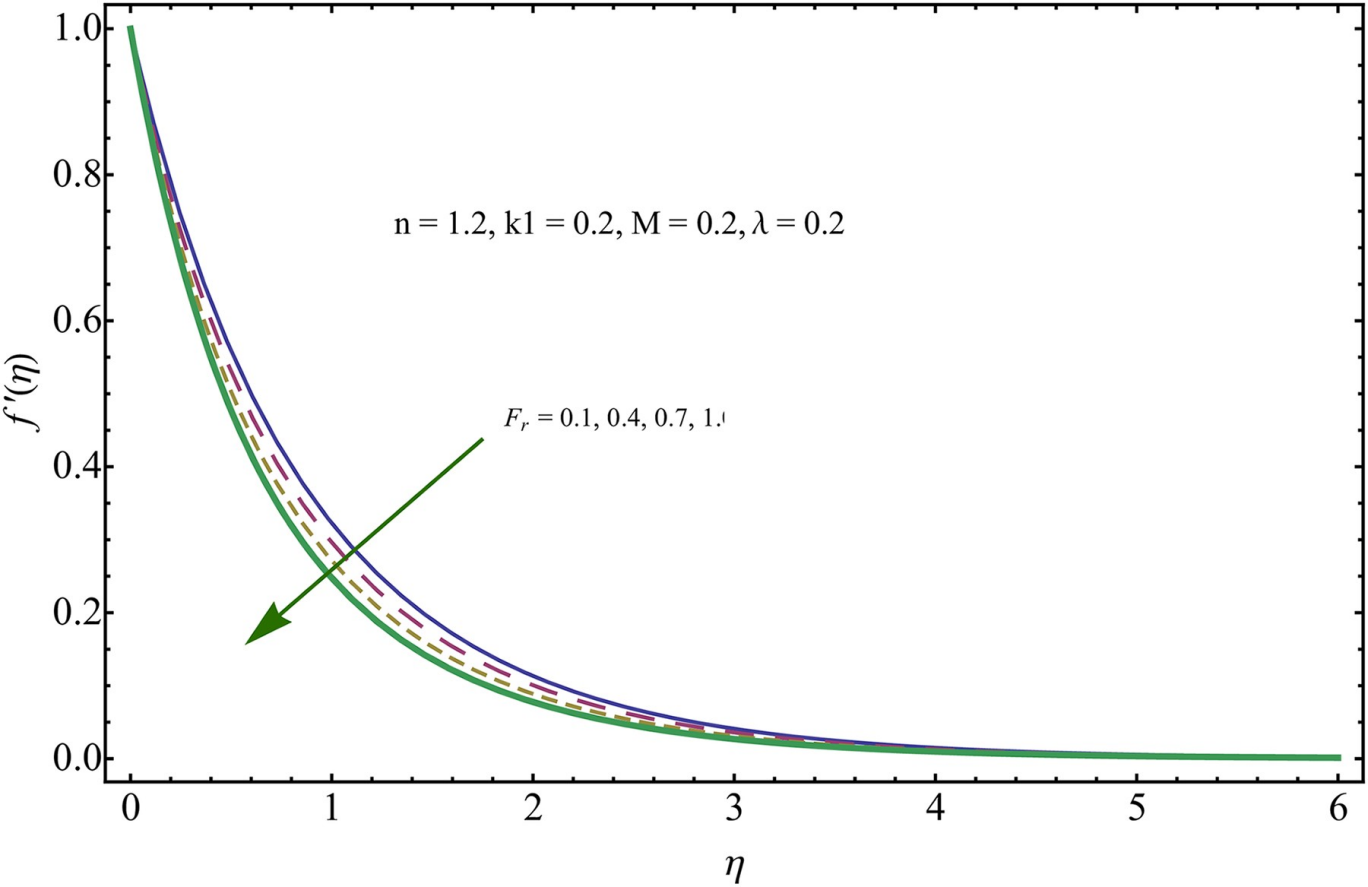
Variation in velocity field for incremental values of *F*_*r*_.

**Fig 5:**
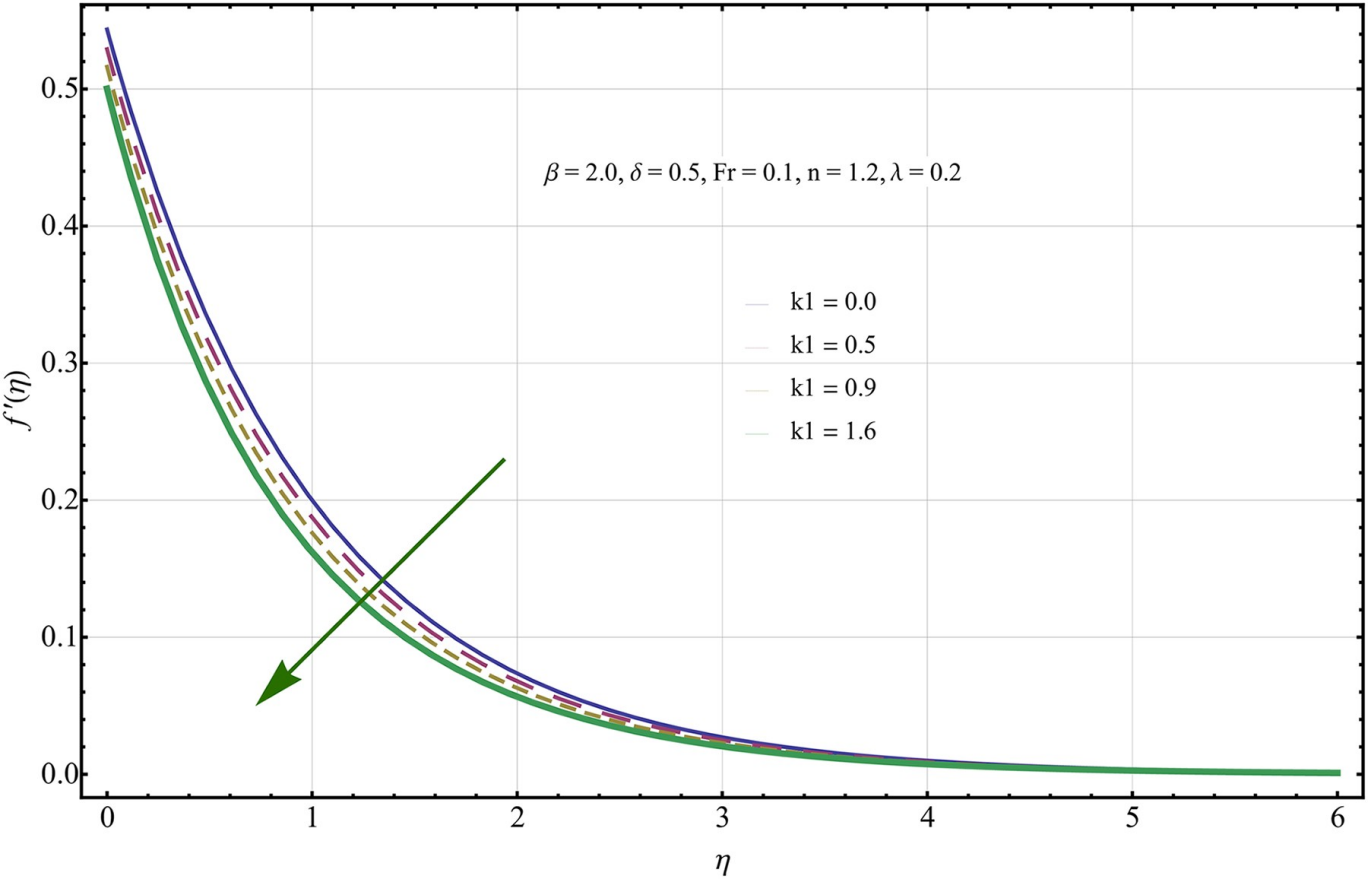
Variation in velocity field for incremental values of *k*_1_.

**Fig 6:**
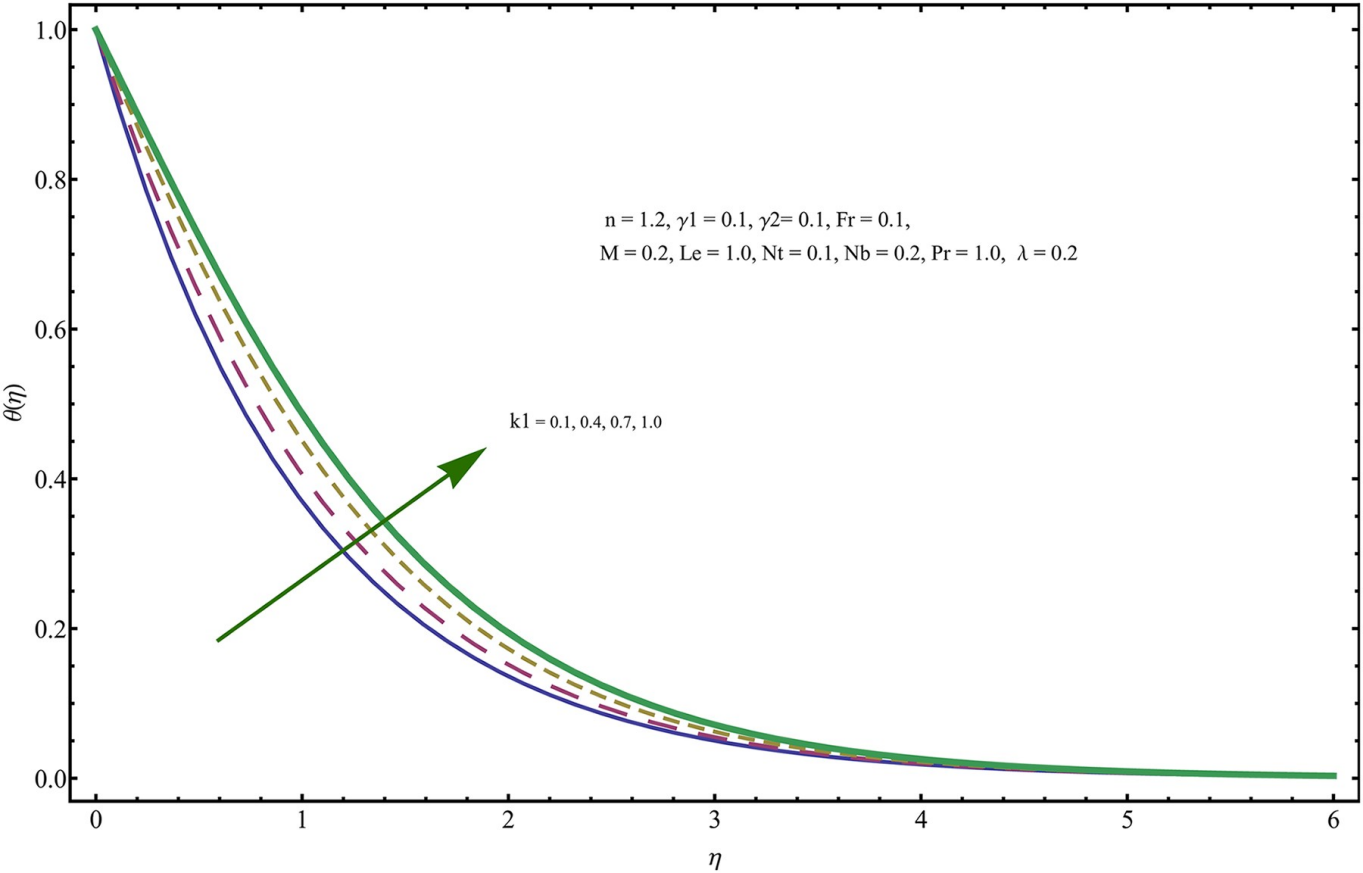
Variation in temperature field for incremental values of *k*_1_.

**Fig 7:**
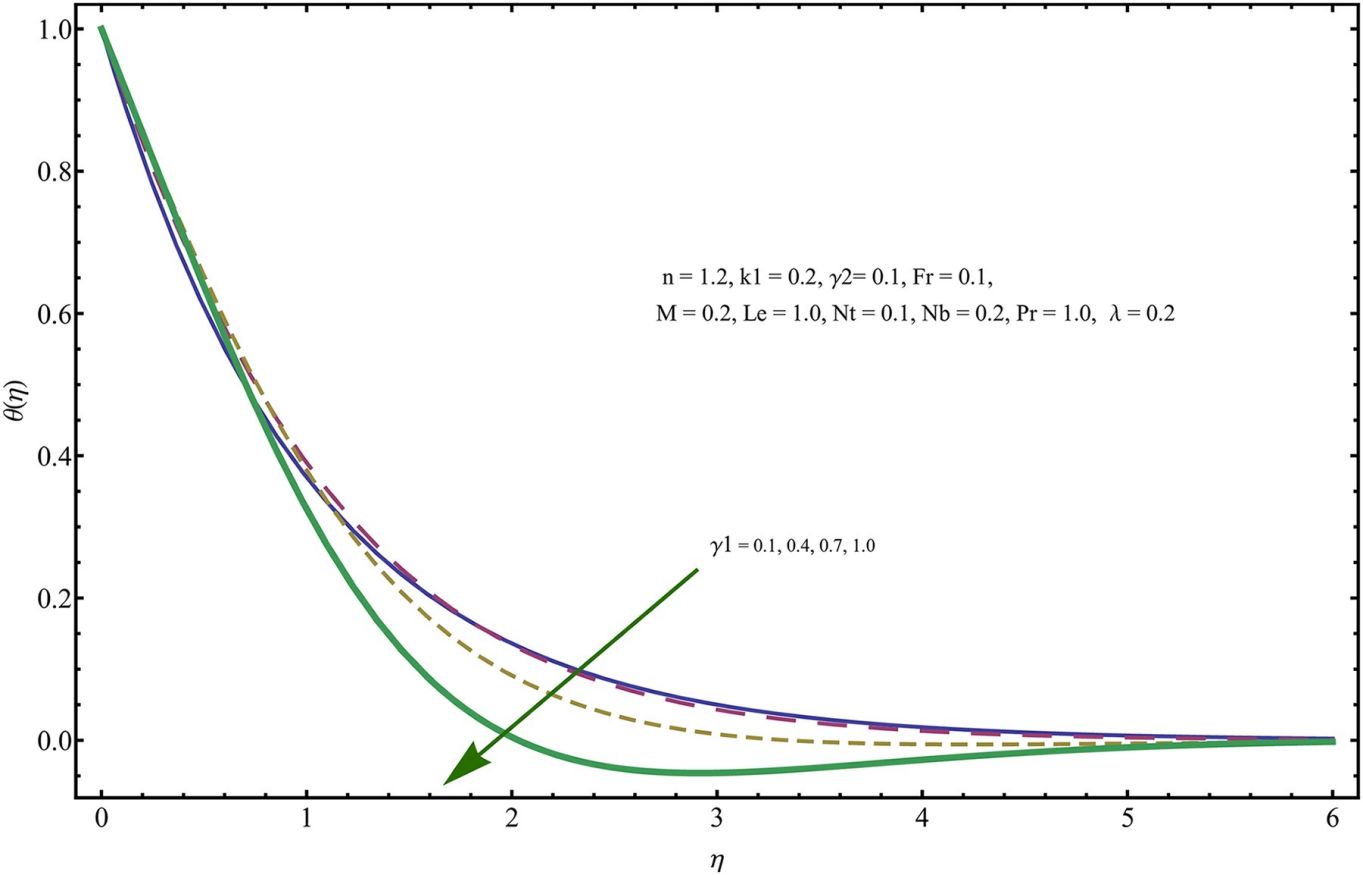
Variation in velocity field for incremental values of *γ*_1_.

**Fig 8:**
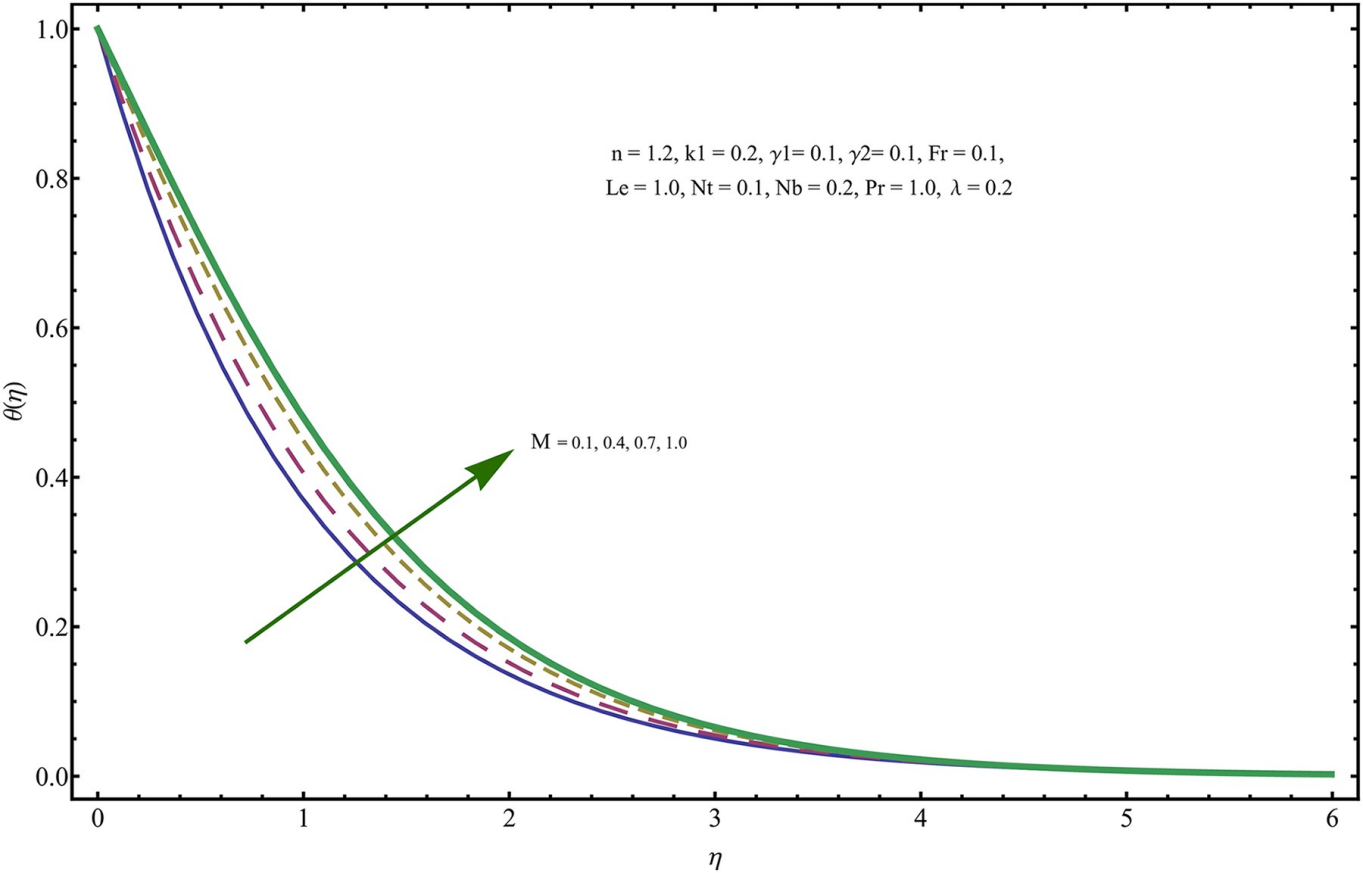
Variation in temperature field for incremental values of *M*.

**Fig 9:**
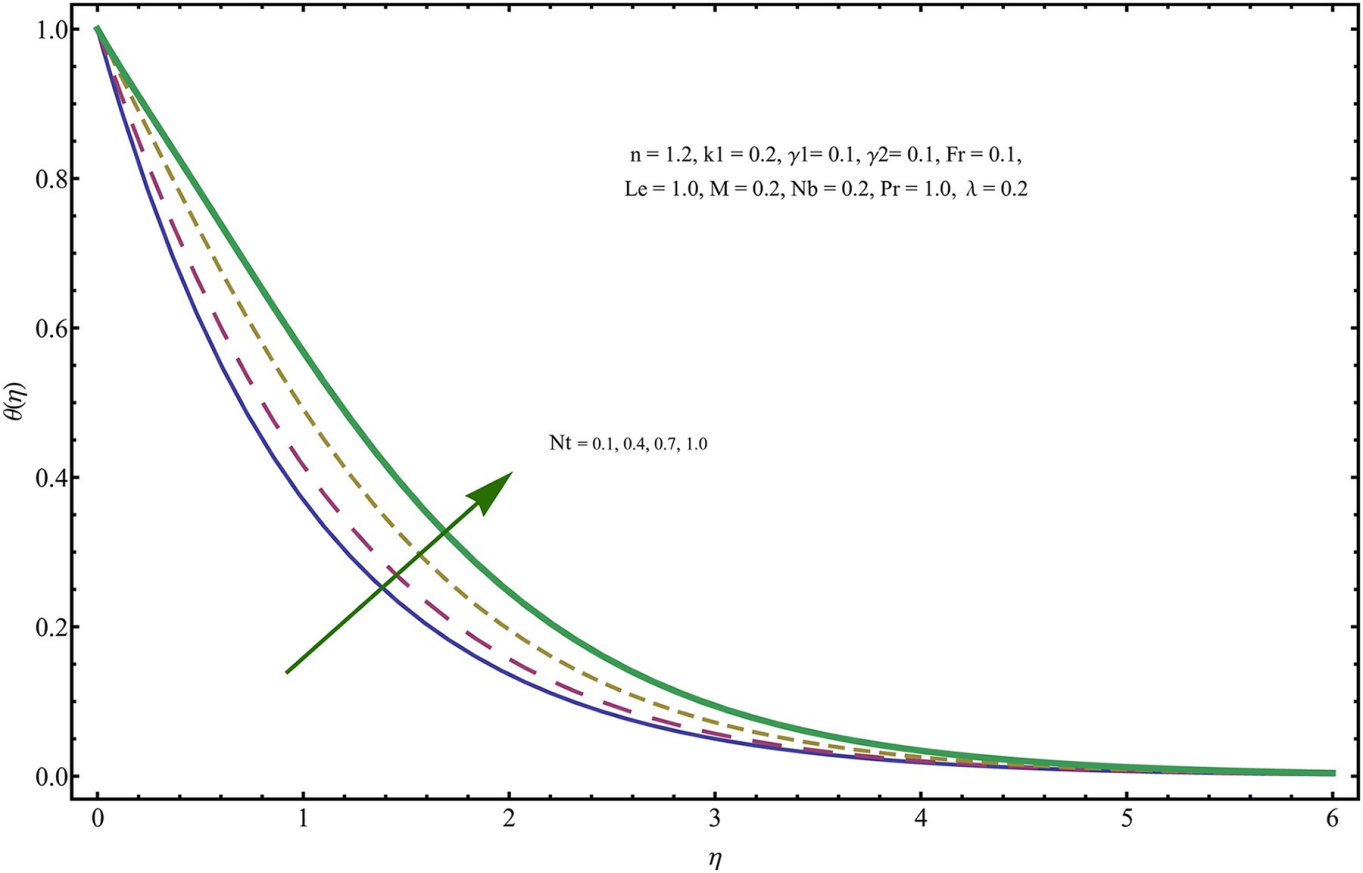
Variation in temperature field for incremental values of *Nt*.

**Fig 10:**
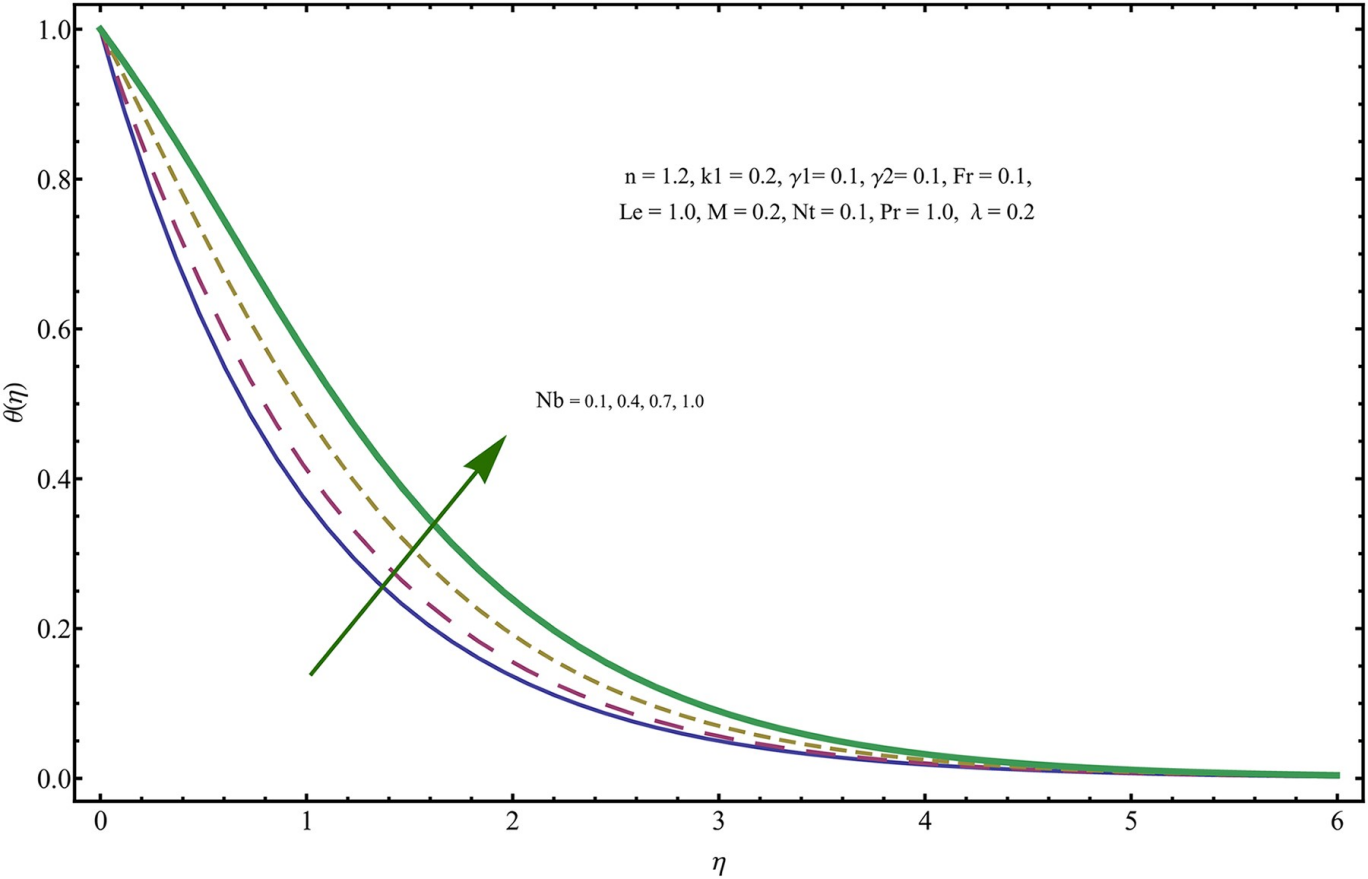
Variation in temperature field for incremental values of *Nb*.

**Fig 11:**
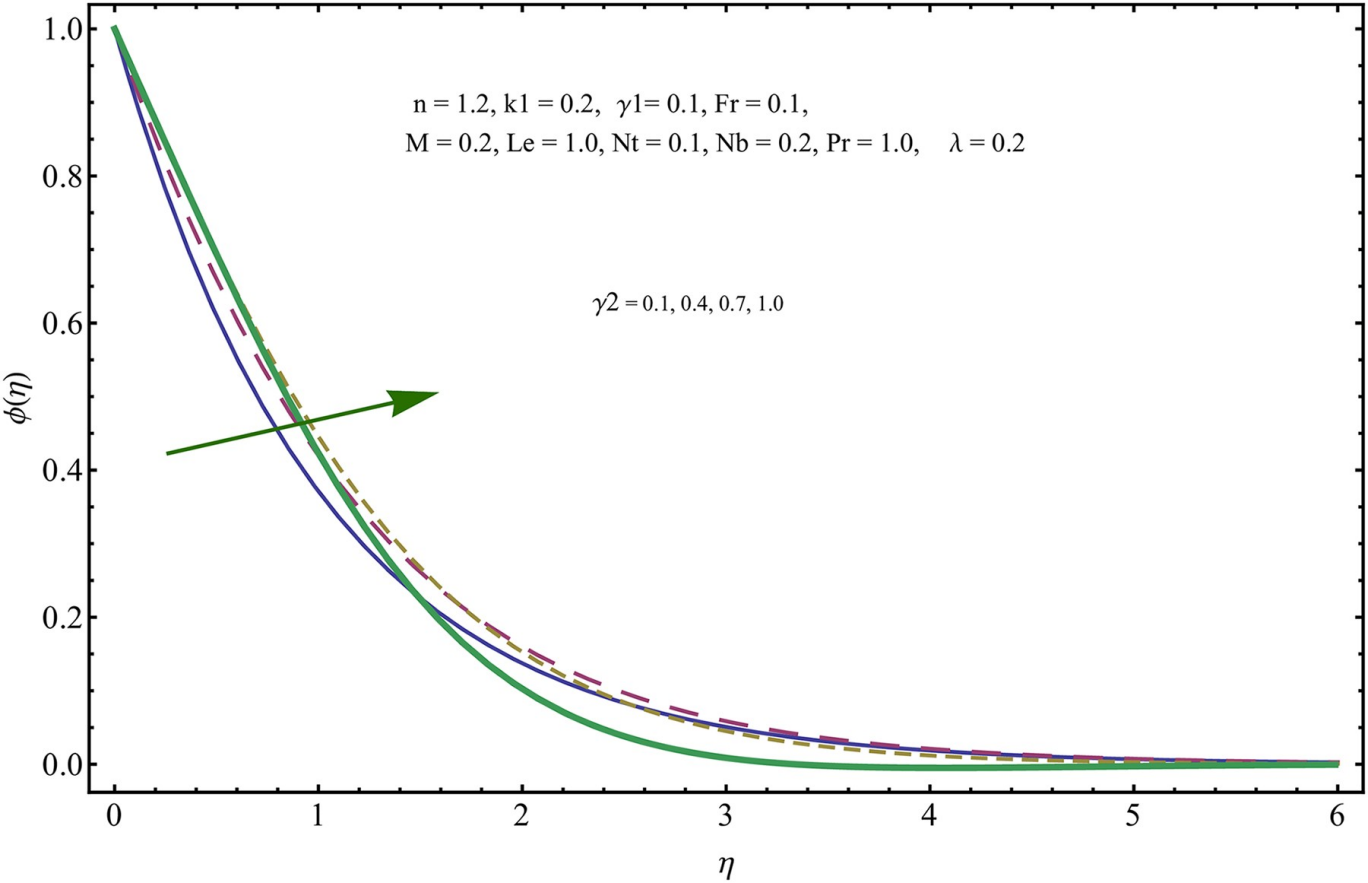
Variation in concentration of nanoparticles for incremental values of *γ*_2_.

**Fig 12:**
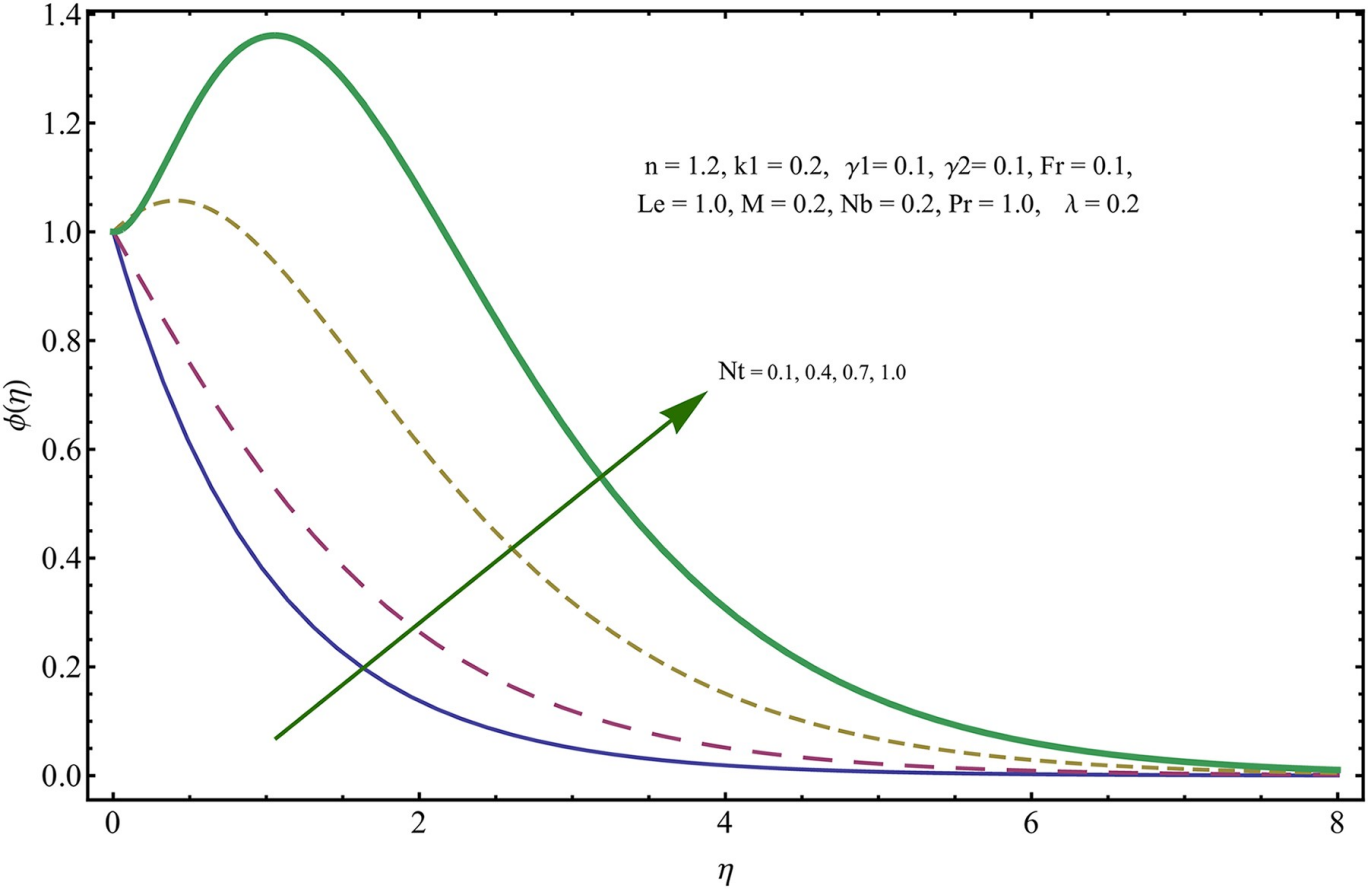
Variation in concentration of nanoparticles for incremental values of *Nt*.

**Fig 13:**
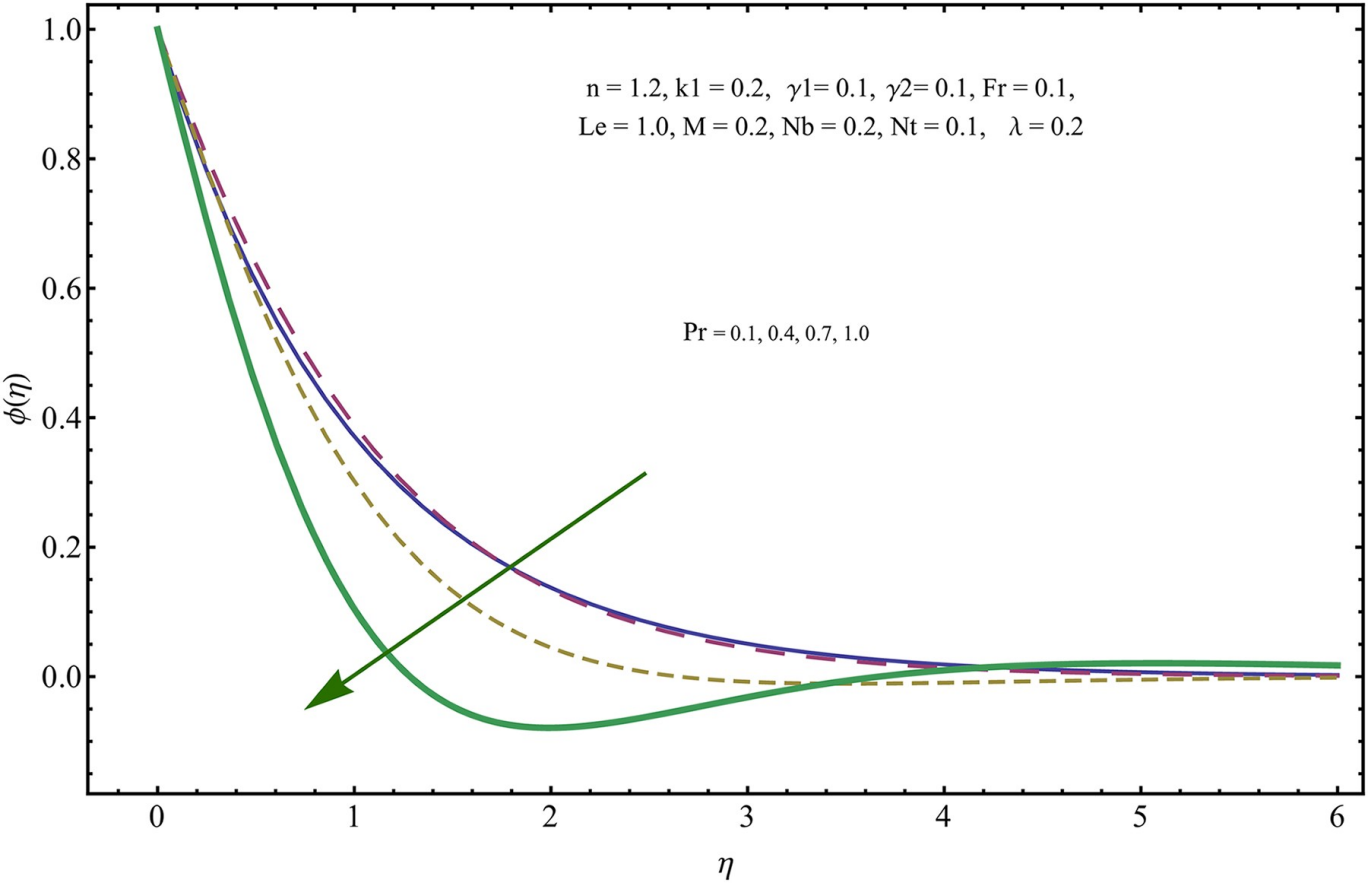
Variation in concentration of nanoparticles for incremental values of *Pr*.

The given non-linear system of Eqs (16)–(18) with boundary conditions (19) is solved by HAM for series solutions. Numerical data for wall-drag (skin-friction) coefficient (*C*_*f*_), local Nusselt (heat flux) (*Nu*) and local Sherwood (mass flux) (*Sh*) is compiled in Tables 2 & 3, respectively. Tables 4 & 5 are the results of comparison/validation of skin friction, local Nusselt and local Sherwood with Rasool et al. [37], respectively. Table 6 is the correlation of the skin-friction while Table 7 is the correlation of local Nusselt and local Sherwood for pertinent fluid parameters. In distinction, a reducing behavior in skin friction (*C*_*f*_) and mass flux (*Sh*) is noted for visco-elastic nanofluid parameter (*k*_1_). Furthermore, the Brownian diffusion rate (*Nb*) and thermophoresis effect (*Nt*) appeared as reducing factors for heat flux (*Nu*). Influence of various fluid parameters on the flow profiles i.e. velocity field, temperature distribution and concentration of the nanoparticles in base conventional fluid, is shown in Figs 3–13. The influences of porosity and inertia on the non-dimensional velocity *f*′(*η*) and corresponding variations are plotted in Figs 3 and 4, respectively. The influence of porosity parameter λ presents a decreasing behavior in the respective profile plotted in Fig 3. Physically, the existence of porous factor results in increment of resistance offered by medium to the fluid motion that causes declination in the fluid momentum and connected boundary layer thickness reduces. Fig 4 shows the change in velocity profile for various incremental values of inertial coefficient. Similar to the porosity factor, reduction in flow profile is witnessed with incremental values of *Fr*. A similar trend is noticed in velocity profile for various values of visco-elastic nano-fluid parameter *k*_1_ plotted in Fig 5. Physically, the incremental values of *k*_1_ result in increasing the fluid viscosity that reduces the flow motion. Figs 6–11. The visco-elastic nanofluid parameter results in increasing thermal field and the associated boundary layer shows increasing thickness. The results are plotted in Fig 6. The relaxation time parameter for the Temperature *γ*_1_ reduces the temperature profile and the associated thickness of thermal boundary layer as well. Physically, it confirms that incremental nature of thermal relaxation time parameter requires more time to shift the heat from intensively packed fluid particles to the low energetic fluid particles. One can call it a demonstration of characteristics of non-conducting fluid material. Thus, a decay in temperature profile is noted. The results are plotted in Fig 7. Fig 8 shows the variation noted in thermal distribution and the associated boundary layer for incremental values of the induced non-uniform magnetic effect. An increasing trend is noticed in temperature profile for stronger magnetic effect. Physically, the retardation offered to the fluid motion by sudden jumps created by Magnetic field increase the particles’ collision which is responsible for increasing trend in temperature boundary layer. Figs 9 and 10 retrieve the increasing trend of Brownian diffusion parameter and thermophoretic force for associated thermal profile. Physically, the erratic motion of fluid packets appeared due to increasing trend in Brownian motion, thus an enhancing nature of temperature profile is noted. Further, the increase in thermophoretic force produces more intensive and vigorous thermophoretic influence causing the nano-particles to move away from the stretching sheet. This development induces boost in the temperature profile. Figs 11–13 are the plots of variation in concentration of nanoparticles against the concentration relaxation time parameters, Thermophoretic force and Prandtl number. Fig 11 is specifically plotted for variation in concentration profile against the incremental values of relaxation parameter for concentration of the nanoparticles. A mixed trend is noted as plotted in the respective figure. Physically, the relaxation parameter allows sufficient time to the nanoparticles to dilute in the base fluid that results in an increasing trend with the passage of time. Fig 12 shows the behavior of concentration profile for incremental values of thermophoretic parameter. An increasing trend is noted for higher values of thermophoresis parameter. Physically, during the thermophoresis, nanoparticles are forced to move from hotter region to the colder region, therefore, the hot particles saturated near the sheet, start moving away from the sheet surface. This movement results in an augmentation in the concentration distribution. The un-deniable fact that the Prandtl number induces a decreasing impact on concentration of nanoparticles is seen in Fig 13. Physically, the incremental values of Prandtl number correspond to a weaker thermal diffusivity. Thus, the concentration of the nanoparticles reduces for higher Prandtl.

**Table 2:** Numerical results/data of skin-friction $\left(\frac{n+1}{2}{)}^{1/2}[(1+\frac{1}{\beta })f^{\prime \prime }\right]$ for both linear and non-linear cases.

*k*_1_	*F*_*r*_	λ	*M*	${Re}_{x}^{1/2}C_{fx}$ (non-linear)	${Re}_{x}^{1/2}C_{fx}$ (linear)
0.0	0.1	0.2	0.2	−0.7706	−0.73600
0.1				−0.55577	−0.53387
0.2				−0.32691	−0.31573
0.3				−0.08405	−0.08160
0.2	0.0	0.2	0.2	−0.31470	−0.30507
	0.3			−0.35131	−0.33707
	0.6			−0.38793	−0.36907
	0.9			−0.42454	−0.40107
0.2	0.1	0.0	0.2	−0.34521	−0.33173
		0.3		−0.31775	−0.30773
		0.6		−0.29029	−0.28373
		0.9		−0.26283	−0.25973
0.2	0.1	0.2	0.0	−0.32019	−0.30933
			0.3	−0.33530	−0.32373
			0.6	−0.38061	−0.36693
			0.9	−0.45612	−0.43893

**Table 3:** Numerical results/data of local Nusselt $\left(-(\frac{n+1}{2}{)}^{1/2}\theta^{\prime }\right)$ and local Sherwood $\left(-(\frac{n+1}{2}{)}^{1/2}\phi^{\prime }\right)$ for both non-linear (n = 1.2) and linear (n = 1) cases at *Pr* = 1, *k*_1_ = 0.2.

*Nb*	*Nt*	*Le*	*γ*_1_	*γ*_2_	$-{Re}_{x}^{-1/2}Nu_{x}$ (non-linear)	$-{Re}_{x}^{-1/2}Sh_{x}$ (non-linear)	$-{Re}_{x}^{-1/2}Nu_{x}$ (linear)	$-{Re}_{x}^{-1/2}Sh_{x}$ (linear)
0.1	0.1	1.0	0.2	0.2	0.6975	0.2884	0.6700	0.2800
0.4	0.1	1.0	0.2	0.2	0.6031	0.6424	0.5800	0.6175
0.7	0.1	1.0	0.2	0.2	0.5087	0.6930	0.4900	0.6677
1.0	0.1	1.0	0.2	0.2	0.4143	0.7132	0.4000	0.6850
0.2	0.1	1.0	0.2	0.2	0.6660	0.5244	0.6400	0.5050
0.2	0.4	1.0	0.2	0.2	0.5663	0.07157	0.5500	0.1700
0.2	0.7	1.0	0.2	0.2	0.4772	0.00615	0.4600	0.0165
0.2	1.0	1.0	0.2	0.2	0.3828	0.00475	0.3700	0.0014
0.2	0.1	0.5	0.2	0.2	0.6660	0.4457	0.6400	0.4300
0.2	0.1	1.0	0.2	0.2	0.6660	0.6031	0.6400	0.5050
0.2	0.1	1.5	0.2	0.2	0.6660	0.6817	0.6400	0.5800
0.2	0.1	2.0	0.2	0.2	0.6660	0.8822	0.6400	0.6550
0.2	0.1	1.0	0.0	0.2	0.6398	0.5244	0.6100	0.5050
0.2	0.1	1.0	0.3	0.2	0.6791	0.5244	0.6550	0.5050
0.2	0.1	1.0	0.6	0.2	0.7521	0.5244	0.7000	0.5050
0.2	0.1	1.0	0.9	0.2	0.8010	0.5244	0.7450	0.5050
0.2	0.1	1.0	0.2	0.0	0.6660	0.4982	0.6400	0.4750
0.2	0.1	1.0	0.2	0.3	0.6660	0.5375	0.6400	0.5200
0.2	0.1	1.0	0.2	0.6	0.6660	0.5865	0.6400	0.5650
0.2	0.1	1.0	0.2	0.9	0.6660	0.6311	0.6400	0.6100

**Table 4:** Comparison of *C*_*f*_ results with Rasool et al. [37].

*F*_*r*_	*C*_*f*_ (Current)	*C*_*f*_ (Rasool et al. [37])
0.0	−1.1899	−1.1950
0.3	−1.2501	−−
0.6	−1.3600	−1.3618
0.9	−1.4314	−−
1.2	−1.5111	−1.5117

**Table 5:** Comparison/validation of results with Rasool et al. [37] setting *γ*_1_ = *γ*_2_ = 0 = *k*_1_, *n* = 1.2.

*N*_*b*_	*N*_*t*_	*Pr*	−*Nu*_*x*_	−*Nu*_*x*_	−*Sh*_*x*_	−*Sh*_*x*_
			(Current)	(Rasool et al. [37])	(Current)	(Rasool et al. [37])
0.1	0.1	1.0	0.4801	−−	0.5001	−−
0.5			0.4312	0.4338	0.5222	0.5227
0.75			0.3722	0.3777	0.5460	0.5478
1.0			0.3200	0.3274	0.5999	0.5600
0.2	0.0	1.0	0.4499	0.4470	0.5790	0.5798
	0.3		0.4091	0.4089	0.4192	0.4198
	0.5		0.3862	0.3858	0.3300	0.3307
	0.7		0.3602	−−	0.2801	−−
0.2	0.1	0.5	0.3222	0.3255	0.2222	0.2217
		1.0	0.5072	0.5086	0.3999	0.4056
		1.5	0.6200	0.6266	0.5684	0.5685
		2.0	0.7101	−−	0.7100	−−

**Table 6:** Correlation of skin-friction (wall-drag).

Parameter	Linear stretching	Nonlinear stretching
*k*_1_	0.9994624	0.9994624
*F*_*r*_	−1	−1
λ	+1	+1
*M*	−0.9583148	−0.9583321

**Table 7:** Correlation of heat and mass flux.

Parameter	Nusselt (n = 1)	Nusselt (n = 2)	Sherwood (n = 1)	Sherwood (n = 2)
*N*_*b*_	+1	+1	−0.8572771	−0.8575619
*N*_*t*_	+1	0.9997769	0.9184755	0.8374697
*Le*	*N*/*A*	*N*/*A*	−1	−0.9873802
*γ*_1_	−1	−0.9939203	*N*/*A*	*N*/*A*
*γ*_2_	*N*/*A*	*N*/*A*	−1	−0.9991548

## Conclusion

A locally similar analysis on Darcy Forchheimer visco-elastic nanofluid flow bounded by a non-linearly stretching sheet/surface manifested with Cattaneo-Christov theory of heat—mass flux has been carried out in this analytic research article. The key findings are itemized below:

- Increasing values of interial coefficient and porosity result in declination of the velocity field and the associated momentum boundary layer.
- The visco-elastic nanofluid parameter shows reduction in the velocity field however, an increment is noted in the thermal layer for augments in the aforementioned parameter.
- The intensive resistance offered by the addition of porosity factor in the flow model results in rise of temperature profile, however, opposite behavior is noticed in concentration of nanoparticles.
- Larger Prandtl number shows reduction in concentration of the nanoparticles.
- Thermal relaxation time parameter allows more heat to be absorbed by the system. Thus, a decay is noted.
- Solute relaxation time parameter shows mixed behavior in concentration of nanoparticles.
- Skin-friction force is intensive for higher thermal relaxation time parameter.
- Local Nusslt shows increasing behavior for higher values of thermal relaxation time parameter, however, the values are higher for non-linear case as compared with linear case.
- Local Sherwood is increasing function of concentration relaxation time parameter, however, the values are lower for linear case as compared to the non-linear case.

**Nomenclature**:

**Table:** 

*u*, *v*	Components of velocity/*m*⋅*s*^−1^
*x*, *y*	Cartesian coordinates/*m*
*μ*	Viscosity (dynamic) of the fluid/*Pa*⋅*s*
*ν*	Viscosity (kinematic) of fluid/*m*^2^⋅*s*^−1^
*B*_0_	Magnetic field/*A*⋅*m*^−1^
*σ*	Electric conductivity/(*Ωm*)^−1^
*K*	Permeability/*H*⋅*m*^−1^
*n*	Positive number
*ρ*_*fl*_	Density/*kg*⋅*m*^−3^
*C*_*b*_	Drag coefficient (dimensionless)
*α*	Thermal diffusivity/*m*^2^⋅*s*^−1^
*k*	Thermal conductivity/*W*⋅*m*^−1^⋅*K*^−1^
*T*_*w*_	Temperature of the wall/*K*
*T*	Temperature/*K*
(*ρ*_*fl*_*c*)_*fl*_	Productive heat capacity (fluid)/*J*⋅*m*^−3^⋅*k*^−1^
(*ρ*_*fl*_*c*)_*np*_	Productive heat capacity (nanoparticles)/*J*⋅*m*^−3^⋅*k*^−1^
*T*_∞_	Temperature away from surface/*K*
*D*_*Th*_	Thermophoretic force effect
*D*_*Br*_	Brownian motion (diffusion)
*M*	Magnetic parameter
*a*	Positive constant number
*F*_*r*_	Local inertia
λ	Porosity
*Le*	Lewis factor
*Pr*	Prandtl factor
*Nt*	Thermophoretic parameter
*Nb*	Brownian diffusion parameter
*Sh*_*x*_	Local Sherwood number (mass flux)
*Nu*_*x*_	Local Nusslt number (heat flux)
*η*	Dimensionless variable
*f*′	Dimensionless velocity
*θ*	Dimensionless temperature field
*ϕ*	Dimensionless concentration of the nanoparticles
*γ*_1_	Thermal relaxation parameter
*γ*_2_	Solute relaxation parameter
